# Case report: AID, a new principle of correction to treat proximal structural curve with a brace

**DOI:** 10.1186/1748-7161-9-S1-P11

**Published:** 2014-12-04

**Authors:** Manuel Rigo

**Affiliations:** 1 Institut Elena Salva, Barcelona, Spain

## Background

There is no wide accepted principle to brace primary thoracic double major and triple structural curve patterns. Axial elongation from a super-structure or three-point system with a neck semi-ring are some of the previously proposed principles.

## Aim

The purpose of this case report is to present a new principle of correction based on axial compression on the convex ribs of the proximal curve.

## Case report

A 14 year old was diagnosed with AIS at 10 years of age. In a first X-ray from February 2010 it was noted a right thoracic curve measuring 7º Cobb, combined with a left lumbar measuring 7º Cobb. Proximal thoracic region was not measurable. One year later, February 2011 the Cobb angles progressed to 18º and 13º respectively, and progression was confirmed on September 2011 with a Cobb angle of 24º in the main thoracic and 26º in the lumbar curve. She was treated with a Boston brace showing poor in-brace correction, with 17º lumbar, 22º main thoracic and 19º proximal curves. In a new X-ray out of brace on May 2012 the angles were 19º, 18º and 21º. With her second Boston brace the values were 19º, 18º and 25º respectively. On December 2012 and with no new reference out-brace the brace was changed to a classical Chêneau type brace, with no in-brace X-ray, mainly due to the over-exposition. Menarche on April 2013. New X-ray on June showed a progression to 21º, 25º and 31º respectively. Due to the bad evolution of the proximal curve we designed a removable superstructure with a combined mechanism: compression on the convex proximal curve and three-point system. After using partial time this super-structure and confirming an acceptable in-brace correction of the proximal curve, her last X-ray on January 2014 showed a stable curve 21º, 27º and 30º respectively, at Risser 3-.

## Discussion

A new principle of correction applicable to the proximal structural curve in thoracic double major and triple structural curves is presented in a single case report. Further research is necessary before to make any conclusion.

## Consent

Written informed consent was obtained from the parents/legal guardian of the patient for publication of this Case report. A copy of the written consent is available for review by the Editor of this journal.

